# The Effects of Preoperative Coronary Collateral Circulation on Cardiac-Related Events after Coronary Artery Bypass Graft Surgery

**DOI:** 10.21470/1678-9741-2019-0375

**Published:** 2021

**Authors:** Hasan Güngör, Fatih Sivri, Burak Oğulcan Yıldırım, Sercan Çayırlı, Özgün Demiroğlu, Cem Utku Yeşilkaya, Cemil Zencir

**Affiliations:** 1 Department of Cardiology, Adnan Menderes University, Aydin, Turkey.

**Keywords:** Coronary Artery Bypass Graft Surgery, Coronary Collateral Circulation, Percutaneous Coronary Intervention, Myocardial Infarction, Heart Failure, Mortality

## Abstract

**Introduction:**

This study aimed to evaluate the effects of coronary collateral circulation (CCC) in patients who had undergone coronary artery bypass grafting (CABG).

**Methods:**

A total of 127 patients who had undergone CABG (2011-2013) were enrolled into this study and follow-up was obtained by phone contact. Patients were categorized into two groups according to preoperative CCC using the Rentrop method. Percutaneous coronary intervention (PCI), recurrent myocardial infarction (MI), stroke, heart failure (HF), and mortality rates were compared between groups. Clinical outcome was defined as combined end point including death, PCI, recurrent MI, stroke, and HF.

**Results:**

Sixty-two of 127 patients had poor CCC and 65 had good CCC. There were no differences in terms of PCI, recurrent MI, and HF between the groups. Stroke (seven of 62 [11.3%] and one of 65 [1.5%], *P*=0.026) and mortality (19 of 62 [30.6%] and 10 of 65 [15.4%], *P*=0.033) rates were significantly higher in poor CCC group than in good CCC group. In Kaplan-Meier analysis, survival time was not statistically different between the groups. Presence of poor CCC resulted in a significantly higher combined end point incidence (*P*=0.011).

**Conclusion:**

Stroke, mortality rates, and combined end point incidence were significantly higher in poor CCC patients than in the good CCC group.

**Table:** 

Abbreviations, acronyms & symbols				
ACE	= Angiotensin-converting enzyme		ICU	= Intensive care unit
AF	= Atrial fibrillation		LA	= Left atrial
BMI	= Body mass index		LDL	= Low-density lipoprotein
CABG	= Coronary artery bypass grafting		LV	= Left ventricular
CAD	= Coronary artery disease		LVEF	= Left ventricular ejection fraction
CC	= Cross-clamp		MI	= Myocardial infarction
CCC	= Coronary collateral circulation		OR	= Odds ratio
CI	= Confidence interval		OS	= Overall survival
COPD	= Chronic obstructive pulmonary disease		PCI	= Percutaneous coronary intervention
DM	= Diabetes mellitus		RCA	= Right coronary artery
HF	= Heart failure		RR	= Relative risk
HT	= Hypertension			

## INTRODUCTION

The myocardial area at risk for infarction, duration of occlusion, absence of coronary collateral circulation (CCC), absence of ischemic preconditioning, and myocardial oxygen consumption during occlusion are the factors which affect the myocardial infarct size^[1,2]^. Theoretically, CCC is an alternative source of blood supply to ischemic myocardium and one of the most important factors of the rate and extent of myocardial cell death^[3]^; however, its prognostic importance for patients with coronary artery disease (CAD) has been still controversial^[4,5]^.

Coronary artery bypass grafting (CABG) is still the standard care for patients with three‐vessel or left main CAD with intermediate or high SYNTAX score (> 22), compared with the percutaneous coronary intervention (PCI)^[6,7]^. The CCC behaves as an alternative source of blood supply in patients with myocardial infarction (MI) and may reduce infarct size and remodel the left ventricle. In literature, there are several publications regarding the benefits of CCC - reducing the tissue injury - in patients with MI^[8-10]^; however, there are limited data on the collateral effects on prognosis and survival in patients who had undergone CABG^[11-13]^. The purpose of this study was to explore the impact of CCC in terms of PCI, recurrent MI, stroke, heart failure, and mortality rates in patients who had undergone CABG.

## METHODS

After approval by the local research ethics committee, the present study was conducted as a follow-up study. In our previous study, we conducted a trial to assess the association between poor CCC and atrial fibrillation (AF) after CABG^[14]^. One hundred sixty-five consecutive patients who were found to have > 95% stenosis in at least one major coronary artery in the coronary angiogram and had undergone CABG between 2011 and 2013 at our department had been included in our previous study. Case selection and exclusion criteria were similar to that described in the authors' previous studies^[14]^. Institutional review board approval was obtained for the present study. By using phone contact, five outcome variables were monitored continuously: PCI, recurrent MI, stroke, heart failure, and mortality rates. In 2019, all of those patients were called by phone, 127 of 165 patients were reached and enrolled into our study.

Details of the baseline clinical characteristics, preoperative treatment, echocardiographic and angiographic findings, and intraoperative and postoperative parameters were recorded. Hypertension was defined as blood pressure > 140/90 mmHg on more than two occasions during office measurements or being on antihypertensive treatment. Diabetes mellitus (DM) was defined as fasting blood glucose of at least 126 mg/dl or being on antidiabetic treatment. Hyperlipidemia was defined as follows: serum low-density lipoprotein cholesterol > 160 mg/dl or total cholesterol > 240 mg/dl or triglyceride > 200 mg/dl or high-density lipoprotein cholesterol < 40 mg/dl or those taking lipid-lowering drugs. Echocardiographic examinations were performed using an iE33 cardiac ultrasound system (Phillips Healthcare, Best, The Netherlands) with 2.5-5-MHz probes. Ejection fraction was calculated using the modified Simpson method.

### Coronary Collateral Scoring

The collateral scoring and the collateral branches evaluation was performed by a single tool developed by Cohen and Rentrop in their study^[15]^. Grades of collateral filling from the contralateral vessel were as follows: 0, none; 1, filling of side branches of the artery to be dilated by collateral channels without visualization of the epicardial segment; 2, partial filling of the epicardial segment by collateral channels; and 3, complete filling of the epicardial segment of the artery being dilated by collateral channels. In patients with more than one collateral vessel supplying the distal aspect of the diseased artery, the highest collateral grade was recorded. Patients were classified according to their CCC grades as either poor (grade 0 or grade 1 collateral circulation) or good (grade 2 or grade 3 collateral circulation)^[16]^.

### Statistical Analysis

The normal distribution of the variables was analyzed by the Kolmogorov-Smirnov test. Normally distributed variables were presented as mean and standard deviation and were compared using the Student's *t*-test, while non‐normally distributed variables were presented as median and its interquartile range and were compared using nonparametric tests such as the Mann‐Whitney two‐sample test. The rate or percentile of the parameters were compared by using Chi-square (χ^2^) test. The presence of the CCC as a dependent factor and the dichotomous variables, PCI, recurrent MI, stroke, heart failure, and mortality rates, were analyzed as covariates for the binary logistic regression models. Overall survival (OS) was calculated through a Kaplan-Meier analysis and was presented as the median and 95% confidence interval (CI). A *P*-value < 0.05 indicated statistical significance. Statistical analysis was performed using the SPSS Inc. Released 2008; SPSS Statistics for Windows, Version 17.0; Chicago: SPSS Inc.

## RESULTS

A total of 127 patients (93 males and 34 females) who had undergone CABG at our department were reached by phone contact and enrolled into our study. Of the patients, 62 had poor CCC and 65 had good CCC. The mean age of the patients was 65.0±9.5 years in the poor CCC group and 62.6±9.9 years in the good CCC group. Hypertension rate, hyperlipidemia rate, smoking, chronic obstructive pulmonary disease rate, pre-treatment use of beta-blockers, angiotensin-converting enzyme inhibitors, calcium channel blockers, and antiplatelet agents were similar among groups. Stroke and DM rates were significantly higher in the poor CCC group than in the good CCC group (seven of 62 [11.3%] and one of 65 [1.5%], *P*=0.030, and 34 of 62 [54.8%] and 25 of 65 [38.5%], *P*=0.047, respectively) (Table 1).

**Table 1:** Preoperative comparison between the patients' clinical features.

Variables	Poor CCC (Rentrop 0-1) n=62	Good CCC (Rentrop 2-3) n=65	*P*-value
			
Age (years)	65.0±9.5	62.6±9.9	0.726
Male, n (%)	44 (71%)	49 (75.4%)	0.689
BMI (kg/m^2^)	29.0±5.9	28.6±4.3	0.130
Follow-up period (years)	6.6±1.7	6.5±1.5	0.885
Stroke, n (%)	7 (11.3)	1 (1.5)	0.030
HT, n (%)	40 (64.5)	44 (67.7)	0.424
Hyperlipidemia, n (%)	17 (27.4)	14 (21.5)	0.286
DM, n (%)	34 (54.8)	25 (38.5)	0.047
Smoking, n (%)	31 (50)	36 (55.4)	0.334
COPD, n (%)	7 (11.3)	6 (9.2)	0.464
β-blockers, n (%)	52 (83.9)	55 (84.6)	0.550
ACE inhibitors, n (%)	32 (51.6)	30 (46.2)	0.331
Calcium channel blockers, n (%)	11 (17.7)	8 (12.3)	0.271
Antiplatelet agents, (%)	17 (27.4)	17 (26.2)	0.515

Data of variables are expressed as mean ± standard deviation (range) or absolute number and its frequencies, n (%) ACE=angiotensin-converting enzyme; BMI=body mass index; CCC=coronary collateral circulation; COPD=chronic obstructive pulmonary disease; DM=diabetes mellitus; HT=hypertension

When we evaluated the echocardiographic parameters of the patients, we found that left atrial size and left ventricular ejection fraction percentile were higher in the poor CCC group than in the good CCC group (3.9±0.4 cm and 3.7±0.3 cm, *P*=0.045, and 55.3±10.6% and 48.1±10.6, *P*=0.003, respectively). Mean left ventricular systolic and diastolic diameters were similar among groups (Table 2).

**Table 2:** Preoperative comparison between the patients' laboratory and echocardiographic parameters.

Variables	Poor CCC (Rentrop 0-1) n=62	Good CCC (Rentrop 2-3) n=65	*P*-value
Blood glucose (mg/dl)	134.3±78.0	131.2±58.8	0.800
Creatinine (mg/dl)	1.2±1.3	0.9±0.3	0.195
Aspartate aminotransferase (IU/l)	33.6±59.0	37.5±39.9	0.662
Alanine aminotransferase (IU/l)	21.1±12.6	31.8±34.8	0.026
Hemoglobin (g/dl)	12.7±1.7	13.5±1.7	0.009
Platelet counts ('10^3^/ml)	261±88	265±99	0.833
LDL (mg/dl)	110.5±30.2	120.0±60.8	0.365
Total cholesterol (mg/dl)	180.4±37.9	191.1±69.2	0.362
Triglyceride (mg/dl)	157.3±84.2	184.4±114.0	0.195
LA size (cm)	3.9±0.4	3.7±0.3	0.045
LV systolic diameter (cm)	3.5±0.6	3.8±0.7	0.056
LV ejection fraction (%)	55.3±10.6	48.1±10.6	0.003
LV diastolic diameter (cm)	6.5±8.5	5.4±0.6	0.418
ICU (hours)	78.4±64.4	60.4±29.3	0.043

Data of variables are expressed as mean ± standard deviation (range) CCC=coronary collateral circulation; ICU=intensive care unit; LA=left atrial; LDL=low-density lipoprotein; LV=Left ventricular

Intraoperative and postoperative data of the patients in the study were depicted in Table 3. Cross-clamp time, number of distal anastomoses, extubation time, and right coronary artery bypass rates were similar among groups (Table 3). When we compared the rates of cardiac-related events in terms of recurrent MI, recurrent PCI, heart failure, stroke, and mortality, we found that stroke and death rates were significantly higher in the poor CCC group than in the good CCC group (seven [11.3%] and one [1.5%], *P*=0.026, and 19 [30.6%] and 10 [15.4%], *P*=0.033, respectively) (Table 4). There were no differences in other cardiac-related events among groups.

**Table 3:** Patients' intraoperative and postoperative data.

Variables	Poor CCC (Rentrop 0-1) n=62	Good CCC (Rentrop 2-3) n=65	*P*-value
			
CC time (min)	52.9±25.4	51.2±18.4	0.635
Number of distal anastomoses (n)	2.9±0.8	2.9±0.7	0.792
Extubation time (h)	20.5±22.7	18.3±17.7	0.543
RCA bypass, n (%)	39 (62.9)	46 (70.8)	0.226

Data of variables are expressed as mean ± standard deviation (range) or absolute number and its frequencies, n (%) CC=cross-clamp; CCC=coronary collateral circulation; RCA=right coronary artery

**Table 4:** Postoperative comparison between the patients' cardiac-related events.

Variables	Poor CCC (Rentrop 0-1)	Good CCC (Rentrop 2-3)	*P*-value
	n=62	n=65	
Recurrent MI, n (%)	2 (3.2)	8 (12.3)	0.056
Recurrent PCI, n (%)	11 (17.7)	6 (9.2)	0.126
Heart failure, n (%)	13 (21.0)	7 (10.8)	0.091
Stroke, n (%)	7 (11.3)	1 (1.5)	0.026
Mortality, n (%)	19 (30.6)	10 (15.4)	0.033
Total	42 (67.7)	20 (30.1)	<0.001

Data of variables are expressed as absolute number and its frequencies, n (%). CCC=coronary collateral circulation; MI=myocardial infarction; PCI=percutaneous coronary intervention; Total=any cardiac-related event

**Fig. 1:**
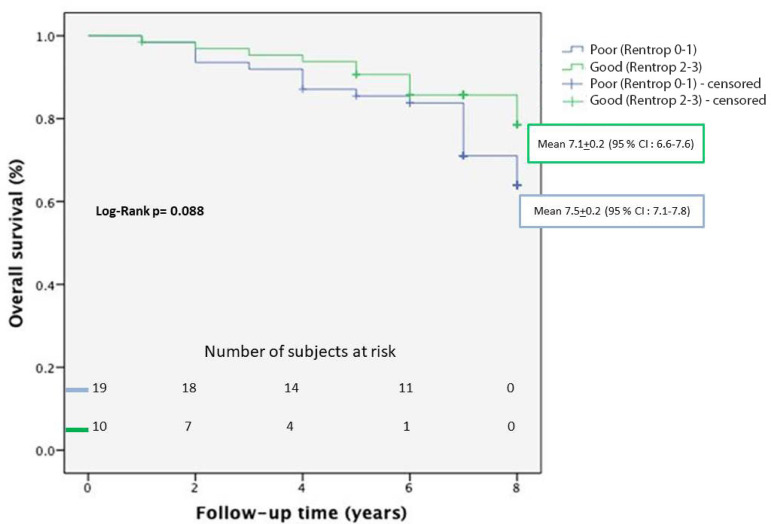
Kaplan-Meier analysis. CI=confidence interval

Univariate logistic regression analyses showed that good CCC significantly decrease the risk of mortality after CABG (odds ratio=0.411, 95% CI=0.174-0.976; *P*=0.044) (Table 5). OS of patients with poor CCC (Rentrop 0-1) and good CCC (Rentrop 2-3) who had undergone CABG was assessed by Kaplan-Meier analysis. In Kaplan-Meier analysis, survival time was not statistically different between the groups (*P*=0.088) (Table 6) (Figure 1). Combined end point incidence of cardiac-related events, including PCI, recurrent MI, stroke, and heart failure, in patients with poor CCC (Rentrop 0-1) and good CCC (Rentrop 2-3) who had undergone CABG were assessed by Kaplan-Meier analysis. Presence of poor coronary collaterals resulted in a significantly higher combined end point incidence in cardiac-related events (*P*=0.011).

**Table 5:** Univariate logistic regression analyses for predicting the effect of good CCC on cardiac-related events after CABG.

		OR 95% (CI)	*P*-value
**Good CCC (Rentrop 2-3)**	Recurrent MI	4.211	0.077
		(0.857-20.675)	
	Recurrent PCI	0.471	0.166
		(0.163-1.365)	
	Heart failure	0.455	0.121
		(0.168-1.230)	
	Stroke	0.123	0.053
		(0.015-1.029)	
	Mortality	0.411	0.044
		(0.174-0.976)	

CABG=coronary artery bypass grafting; CCC=coronary collateral circulation; CI=confidence interval; MI=myocardial infarction; OR=odds ratio; PCI=percutaneous coronary intervention

**Table 6:** Overall survival of patients with poor CCC (Rentrop 0-1) and good CCC (Rentrop 2-3) who had undergone CABG as assessed by Kaplan-Meier analysis.

Variables	Poor CCC (Rentrop 0-1) n=62	Good CCC (Rentrop 2-3) n=65	*P*-value
Mean survival (years)	7.5±0.2	7.1±0.2	0.088
CI (95%)	7.1-7.8	6.6-7.6	

CABG=coronary artery bypass grafting; CCC=coronary collateral circulation; CI=confidence interval

## DISCUSSION

In the current study, we found that stroke and mortality rates were significantly higher in the poor CCC group than in the good CCC group. Patients with good CCC had longer survival time than patients with poor CCC, however this difference did not yield a significantly better median OS.

Theoretically, CCC is an important alternative blood supply in case of MI^[3]^. However, it is still not well known how this protective mechanism works^[4,5]^. Acute MI leads to longer QT interval and this situation increases the risk of arrhythmias. CCC may reduce the QT interval and the risk of arrhythmias^[17]^. In our previous study, we found that patients with poor CCC had a higher risk for AF after CABG^[14]^. We speculated that good CCC may affect the occurrence of AF after CABG by reducing atrial ischemia, oxidative damage, inflammation, fibrosis, lipid deposition, and dilatation^[14]^. Also, CCC has positive clinical effects through smaller infarct size and reduction in post-infarct ventricular dilatation. All those described prognostic factors should reduce the cardiac-related event rate and have clinical beneficial effects on reducing mortality^[18]^.

In a meta-analysis, it was reported that patients with a high collateralization had a significantly reduced mortality risk compared with patients with low collateralization (relative risk [RR]=0.64)^[5]^. Those results were comparable with our study. In our study, we showed that good CCC reduced the mortality risk in patients who had undergone CABG (RR=0.411). However, in the current study, we found that left ventricular ejection fraction (LVEF) was significantly lower in the good CCC group than in the poor CCC group (48.1±10.6% *vs*. 55.3±10.6%, *P*=0.003, respectively). This is an interesting finding, because we expect that good CCC prevents the myocardium from ischemia and tissue injury; consequently, it provides higher LVEF as it was shown in previous studies^[2,3]^. However, similarly to our study, Caputo et al.^[13]^ and Regieli et al.^[19]^ have found that patients with good CCC had lower LVEF than patients with poor CCC. The development of CCC should be considered as a result of angina and more severe and extensive myocardial ischemia of multivessel disease^[20]^.

Tatli et al.^[12]^ compared medical therapy and CABG in patients with good CCC and they reported that revascularization did not affect mortality in patients with CCC. The follow-up period was five years and the rate of survival did not differ among groups in that study. In an another study comparing the effects of CCC after CABG, survival rate in five years was similar between the poor and good CCC groups (84.8% in the CCC group and 89.2% in the no-CCC group)^[13]^. Cardiac-related event-free survival after five years was 50.6% in the CCC group and 54.5% in the no-CCC group with no significant differences between both groups, as well^[13]^. However, in the present study, survival rate was found to be significantly higher in the good CCC group than in the poor CCC group (84.6% and 69.4%, respectively), and cardiac-related event-free survival was significantly higher in the good CCC group than in the poor CCC group (69.9% and 32.3%, respectively).

In our study, we found that stroke rate was significantly higher in the poor CCC group than in the good CCC group (seven of 62 [11.3%] vs. one of 65 [1.5 %], respectively). This finding may be associated with the AF rate in the study population. As previously reported, AF was associated with high stroke incidence rate^[21]^. In our previous study including the similar patient population (we could not reach all the patients at the follow-up period), we reported that the AF rate in the poor CCC group was significantly higher than in the good CCC group (37 of 76 [49%] vs. 12 of 89 [14%], P<0.001, respectively)^[14]^.

Combined end point incidence of cardiac-related events, including PCI, recurrent MI, stroke, and heart failure, in patients with poor CCC (Rentrop 0-1) and good CCC (Rentrop 2-3) who had undergone CABG were assessed by Kaplan-Meier analysis. Presence of poor coronary collaterals resulted in a significantly higher combined end point incidence in cardiac-related events (*P*=0.011). In the present study, the follow-up period was 6.6±1.7 years in the poor CCC group and 6.5±1.5 years in the good CCC group. The follow-up period should be considered relatively short. In our opinion, higher incidence of cardiac-related events in patients with poor CCC may result in increased mortality rate in a longer follow-up period in those patients.

In the present study, we found that recurrent MI rate was higher in the good CCC group than in the poor CCC group (eight of 65 [12.3%] *vs*. two of 62 [3.2%], *P*=0.056, respectively), but it did not reach a statistically significant value. Our findings are consistent with the competition theory^[3]^. Good CCC may affect negatively coronary arteries such as coronary steal during myocardial hyperaemia by competing antegrade flow^[3]^. This situation may increase the risk of restenosis by reducing the flow velocity at the ischemic field with augmented platelet adherence, thrombus formation, and endothelial proliferation^[3]^.

### Limitations

The major limitations of the current study were the relatively small number of patients and the loss of follow-up of almost 40 patients, which can affect the results.

## CONCLUSION

We found that patients with poor CCC may have a higher risk for cardiac-related events including stroke and mortality after CABG surgery. Although there were no significant differences for OS between the poor and good CCC groups, the patients with good CCC had longer survival time than the patients with poor CCC. Further prospective, randomized, controlled trials are needed to confirm the effects of CCC on cardiac-related events.

**Table:** 

Authors' roles & responsibilities	
HG	Substantial contributions to the conception or design of the work; and analysis of data for the work; drafting the work or revising it critically for important intellectual content; final approval of the version to be published
FS	Substantial contributions to the conception or design of the work; final approval of the version to be published
BOY	Analysis of data for the work; final approval of the version to be published
SC	Analysis of data for the work; final approval of the version to be published
OD	Interpretation of data for the work; final approval of the version to be published
CUY	Substantial contributions to the conception or design of the work; final approval of the version to be published
CZ	Acquisition of data for the work; final approval of the version to be published
